# Supplementary material for: Uncertainty and reward histories have distinct effects on decisions after wins and losses
Source: Sci Rep. 2026 Jan 31;16:6795. doi: 10.1038/s41598-026-37554-3 (PMC12916781; doi:10.1038/s41598-026-37554-3)
Supplement: Supplementary file 1 — Supplementary Material 1 [file 41598_2026_37554_MOESM1_ESM.docx]

**Supplementary Materials**





***Figure S1.*** *Asymmetrical learning strategies in the probabilistic reversal learning task.* a) rats had a higher WS probability compared to the LS probability in the PRL task (main effect; F(1,52)=1076.6; p = 2.2e-16). There was an interaction between the WSLS factor and sex (F(1,52) = 41.83, p = 3.5e-08). Subsequent t-tests revealed that, females had a lower WS probability, but a higher LS probability, compared to males. **b)** asymmetry emerges for WS and LS behaviors (main effect of WSLS factor; F(1,26) = 66.55, p = 1.2e-08), where both males and females WS more in the late phase of the block, compared to the early phase. However, only males developed the strategy of also reducing their LS probability in the late phase of the block (p = 0.002, Cohen’s d = 1.34). **c-d)** There were differences in WS and LS probabilities between phases (interaction between WSLS type and phase type; F(1,104) = 198.86, p = 2.2e-16). Subsequent t-tests revealed a significant difference in early versus late phase WS (p < 1e-04, Cohen’s d = 2.80) and LS probabilities (p < 1e-04, Cohen’s d = 2.76). In the late phase, rats were more likely to WS if that win was from the better action, compared to the worse action. Conversely, rats were less likely to LS if that loss was from this better action, compared to the worse action, which emerged in the late phase of the block.


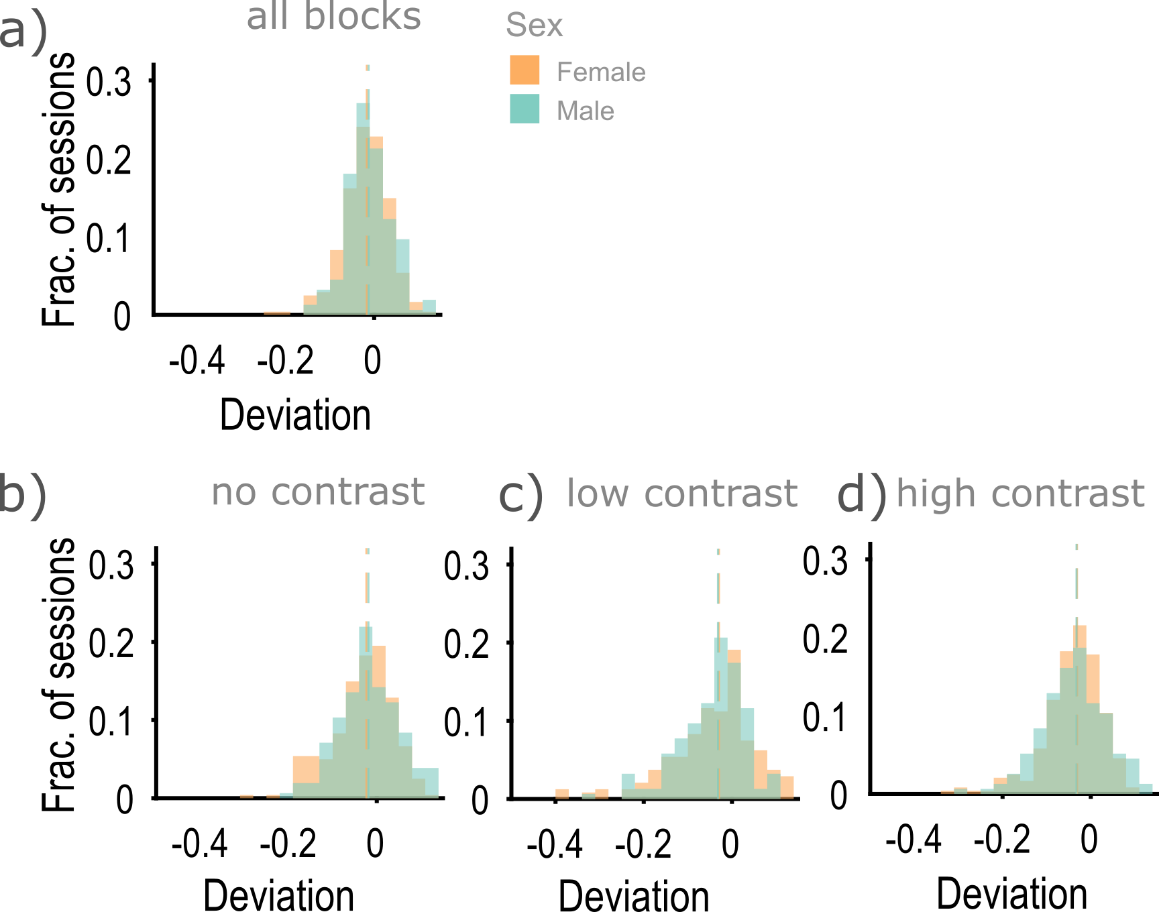


***Figure S2.*** *Males and females deviate at similar levels from the matching behaviors.* **a)** Both sex groups have a higher matching score (i.e., a higher deviation of choice probability from the reward probability based on the choice). However, in across all blocks **(a),** and within the three block types **(b-d)**, there were no sex differences, indicating that males and females make similar choices based on their local reward outcomes.


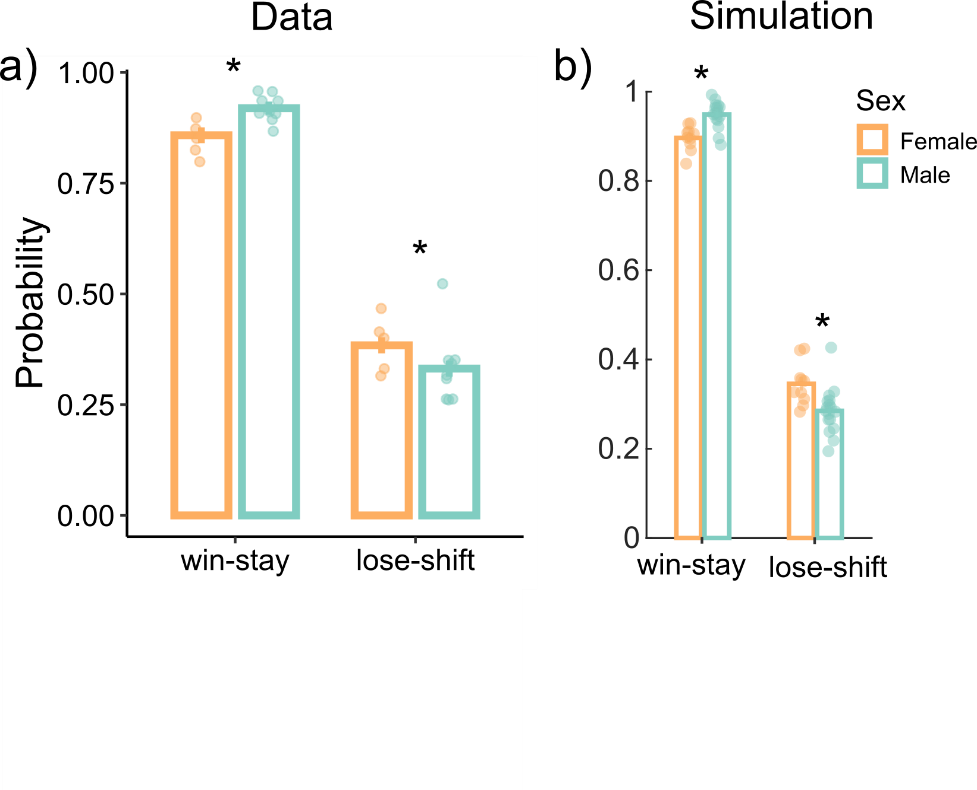


***Figure S3.*** *The estimated asymmetrical learning model parameters can capture the overall win-stay and lose-shift data.* **a)** The overall win-stay (WS) and lose-shift (LS) data from Figure 1d, main text. **b)** Simulated data using the recovered parameter estimates from each rat. The simulation captures the overall sex differences in WS/LS behaviors found in the data (win-stay, p = 0.0001 and lose-shift, p = 0.0054). Each agent was run on the dynaPRL task 50 times (emulating sessions) for 500 trials each session.

**Latency data:**

Latency data were analyzed at three different time points within the trial (see Figure S4a). The latency data was log-transformed (natural log) and no exclusions were made. We log-transformed to reduce skewness, making it more suitable for statistical tests. The smaller the number (including more negative), the faster the latency. The first interval of interest was trial initiation latency, which captured the time between the onset of a new trial and when the rat entered the port to initiate the trial. Faster latency times here reflect higher motivation to start the trial. Overall, we found that males were faster at initiating trials compared to females (Figure S4b; p = 0.005). We further split these latency times conditional on whether the previous trial was a win or a loss. Both males and females were faster to initiate the trial when the previous trial was a win (p = 1.82e-30 and 2.06e-42 for males and females, respectively). This result indicated that a win motivated both sex groups to initiate the next trial faster. Further, males were significantly faster than females to initiate the next trial if the previous trial was a loss (p = 0.001), however, there were no sex differences when the previous trial was a win (p = 0.12). Collectively, these results suggested males may have higher motivation to initiate the next trial compared to females when the previous trial was a loss.

Choice latency was analyzed next, which was the time from levers insertion until a choice was made by pressing one of the two levers (Figure S4a). Overall, we found that males were faster than females at making a choice, although this was a weak effect (Figure S4d; p = 0.0495). Next, the choice latency data were split based on whether the current trial was a stay choice or a switch choice, relative to the previous (e.g., if the previous trial was a left lever press, and the current trial was also a left lever press, this counted as a stay trial, and latency of the current trial was analyzed). Both males and females were faster when the current trial was a stay trial (p = 9.86e-07 and p = 2.3e-30, for females and males, respectively). Therefore, as expected, a stay choice was faster than a switch choice. Further, we found a significant interaction between trial type (stay or switch) and sex (F(1,788) = 6.34, p=0.012), however, we did not find significant sex differences based on post-hoc tests with Bonferroni correction.

Lastly, we analyzed port entry latency data (Figure S4a), which reflected the latency following the choice (lever press) to entering the port where reward is delivered (if it was a win). We did not find a significant difference between sex groups when analyzing overall latency (Figure S4f; p = 0.61), and also when splitting this measure based on whether it was a win or a loss trial (main effect of group F(1,788) = 0.24, p = 0.63). There was also no main effect of trial type (win or loss; F(1,788) = 2.12, p = 0.13), indicating that irrespective of a win or a loss, both sex groups checked the port at similar latencies.


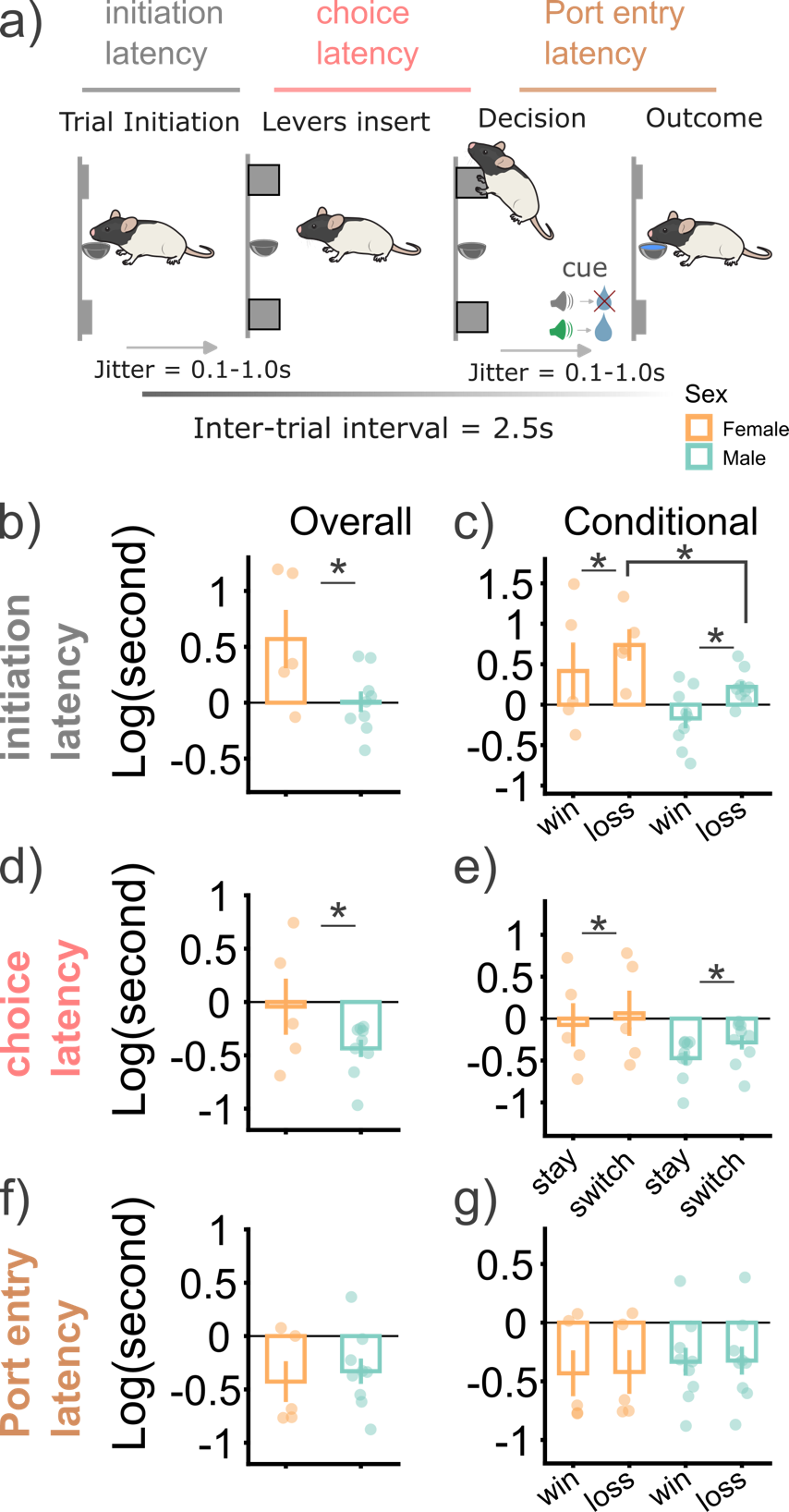


**Figure S4.** *Latency data.* **a)** the three timepoints within a trial where latency data were analyzed. The first timepoint is latency to initiate a trial. The second is choice latency following lever insertion. The third is port entry latency, which is the time between the choice and entry to the port where reward is collected. **b)** Males were overall faster than females to initiate a trial. **c)** Both males and females were faster to initiate the trial when the previous trial was a win compared to a loss. Additionally, males were faster than females when the previous trial was a loss, with no significant differences when the previous trial was a win. **d)** Males were faster than females when making a choice. **e)** Both males and females were faster at making a choice when it was the same as the previous trial (stay trial) compared to when the choice was different (switch trial). **f)** there were no sex differences in the latency to enter the reward port, and **g)** splitting trials based on whether it was a win or a loss trial did not influence this latency.

**Comparing win-stay and lose-stay probabilities:**

While we focused on win-stay and lose-shift behaviors to understand sensitivity to wins and losses, these measures could be biased by overall differences in the stay and switch probabilities, irrespective of the outcome. For example, a high win-stay and low lose-shift probability could simply manifest as a high stay probability irrespective of the outcome. To further investigate whether rats were sensitive to outcomes, we compared win-stay and lose-stay probabilities. Overall (across all block and action types), rats had a higher win-stay probability compared to lose-stay (Figure S5a), suggesting that they were sensitive to the outcomes when making stay decisions (i.e., they were more likely to stay following a win, and less likely to stay following a loss; main effect of wins-stay/lose-stay factor: p = 2.2e-16). Further, male rats were more likely to win-stay (p = 0.0001) and lose-stay (p = 0.0001) compared to females, suggesting that irrespective of the outcome, males had a higher stay probability compared to females (Figure S5a). We also compared win-stay and lose-stay probabilities across block and action types in the late phase (Figure S5b-e). Across all these subdivisions of block and action types, rats were overall more likely to win-stay compared to lose-stay (statistics reported in figure legend) suggesting sensitivity to the outcome when making stay decisions.


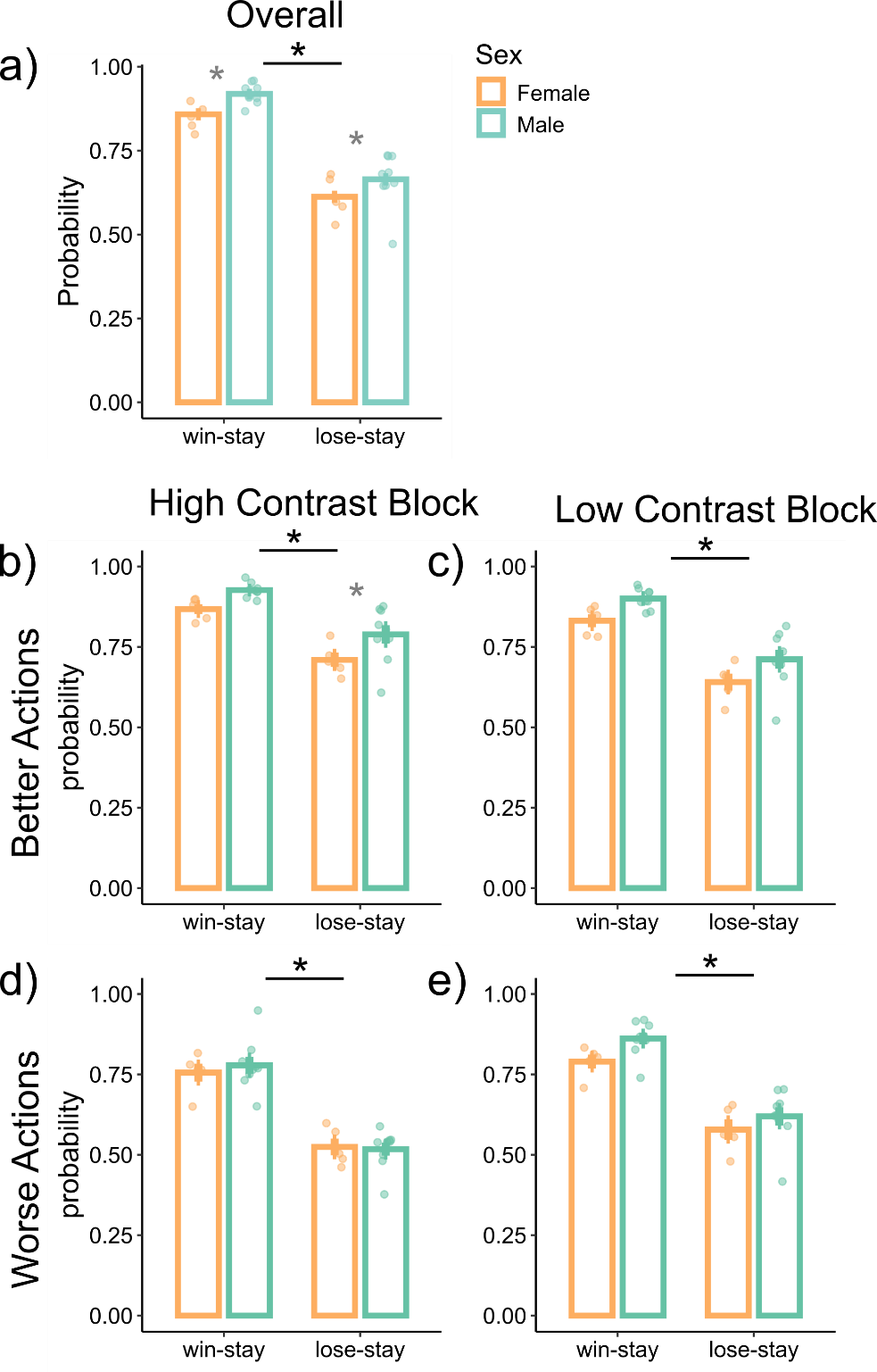


**Figure S5.** *Comparison of win-stay and lose-stay probabilities.* **a)** Overall (across all block types and action types), rats were more likely to win-stay than they were to lose-stay, suggesting sensitivity to the outcome when making stay decisions. **b)** Comparison of win and loss stay probabilities in the high contrast block for better actions. There was a significant main effect of win and lose stay (WLstay; F(1,12) = 58.86, p = 5.75e-06), and a significant main effect of sex group (F(1,12) = 7.43, p = 0.018). Males were significantly more likely to lose-stay than females (p = 0.021), but not win-stay (p = 0.07). **c)** Comparison of win and loss stay for better actions in the low contrast block. There was a significant main effect of WLstay factor (F(1,12) = 87.94, p = 7.14e-07), and a significant main effect of sex (F(1,12) = 6.4, p = 0.023), with a non-significant trend of males more likely to win-stay (p = 0.056) and lose-stay (p = 0.051). **d)** Comparison of win and loss stay for worse actions in the high contrast block. There was a significant main effect of WLstay factor (F(1,12) = 85.15, p = 2.31e-09), and no significant main effect of sex (F(1,12) = 0.091, p = 0.77). **e)** Comparison of win and loss stay for worse actions in the low contrast block. There was a significant main effect of WLstay factor (F(1,12) = 206.85, p = 6.28e-09), and no significant main effect of sex (F(1,12) = 2.63, p = 0.13). Asterix symbol with bar underneath are significance on main effect of WLstay factor. Asterix symbol with no bar are sex differences.

**Combining win-stay and lose-shift probabilities for information theory analysis:**

Here we combined win-stay and lose-shift probabilities into a single metric reflecting reward dependent strategies. This was based on previous work^1,2^, defining this metric as entropy of reward-dependent strategy (ERDS). The ERDS is calculated as the following:

$ERDS= -(P(win) \times WS \times log₂(WS) + P(win) \times(1 - WS) \times log₂(1 - WS) + (1 - P(win)) \times(1 - LS) \times log₂(1 - LS) + (1 - P(win)) \times LS \times log₂(LS))$

Where p(win) is the probability of win, and WS is the probability of win-stay and LS the probability of lose-shift. Overall, a lower ERDS score reflects a more consistent (or predictable) response strategy to reward-based feedback. Also, the p(win) influences the ERDS calculation, where a lower p(win) would decrease the influence of WS on ERDS, as a very low number of wins are less relevant in predicting reward-dependent strategies. And similarly, as the p(win) increases, the influence of LS on ERDS decreases as losses are less frequently and therefore have less explanatory power in predicting behavior.

Males had a lower ERDS score than females (Figure S6a) across all block and action types (p = 0.0043). Further, in the high (Figure S6b) and low contrast blocks (Figure S6c), worse actions had a higher ERDS score than better actions (p < 0.0001), suggesting that better actions reflect a more consistent and predictable reward dependent strategy. Further, males had a significantly lower ERDS score for better actions in low (p = 0.0007) and high contrast blocks (p < 0.0001), with both groups having a similar ERDS scores for worse actions (p > 0.05). In sum, males had a lower ERDS score reflecting a more consistent reward-dependent strategy compared to females.


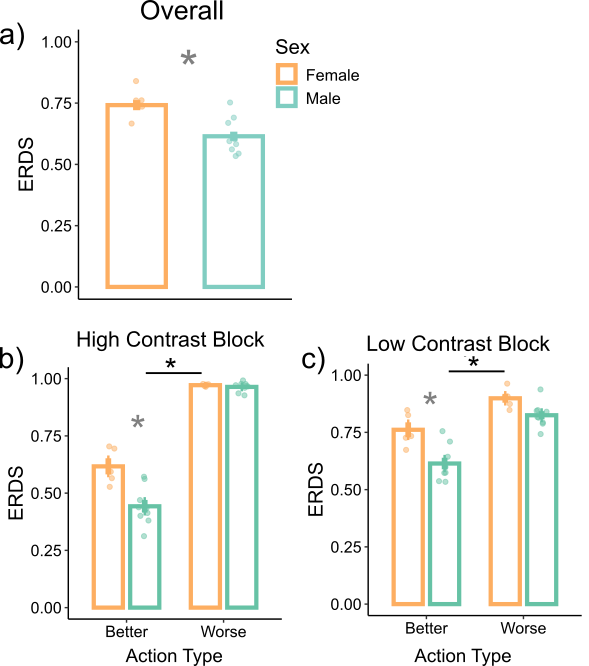


**Figure S6.** *Information theory analyses calculating entropy of reward-dependent strategy (ERDS).* **a)** Across all trial and action types, males had a lower ERDS score compared to females. **b)** In the high contrast block, males had a lower ERDS score for better actions, with no sex differences for worse actions. Further, worse actions overall had a higher ERDS score. **c)** In the low contrast block, males had a lower ERDS score than females for better actions, with no differences for the worse action. Also consistent with the high contrast block, the low contrast block had a higher ERDS score for worse actions overall, compared to the better actions.


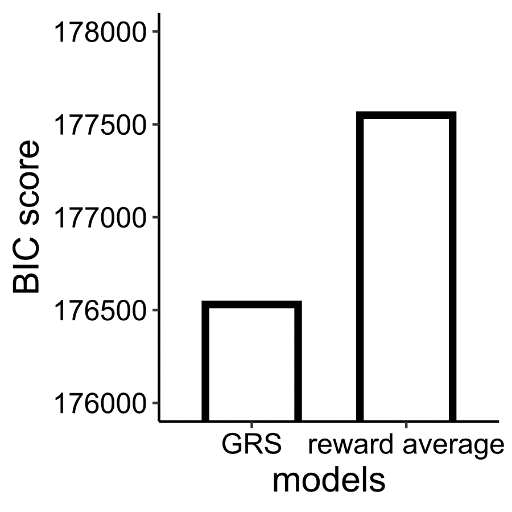


***Figure S7.*** *Model comparison between standard reward average and global reward state (GRS).* Based on Bayesian Information Criterion (BIC) score, where a lower value indicates a better fit with the data. The GRS model fit the data better than a standard reward average model where the current and previous reward was averaged.


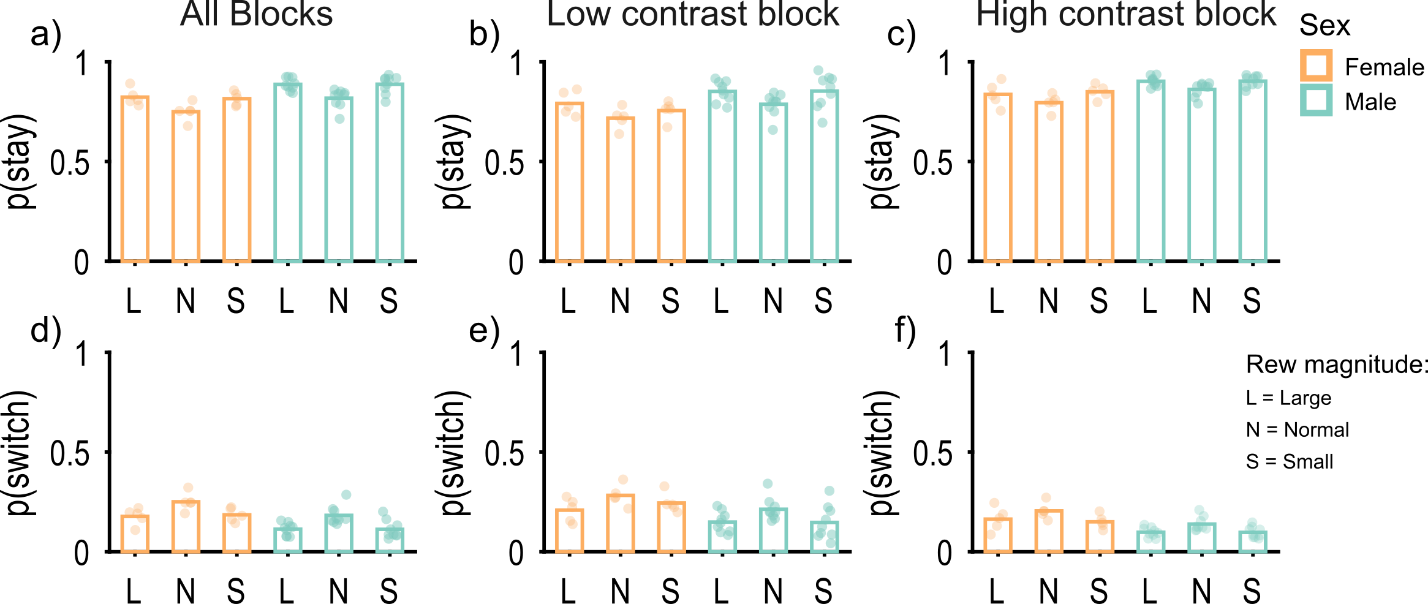


**Figure S8.** *The effect of reward magnitude manipulation on stay and switch behaviors.* Data shown represent the mean (+/-_SEM) stay (upper graphs) and switch (lower graphs) probability for male and female rats exposed to multiple reward magnitudes during testing. There were no significant main effects of reward magnitude manipulation on the probability of stay and switch behaviors, when analyzed across all blocks and separately per block type. Normal magnitude was 33μL, large was 66μL and small was 16.5μL. a) Main effect of reward magnitude manipulation F(1,1172) = 0.31, p = 0.58. b) Main effect of reward magnitude manipulation F(1,1145) = 1.85, p = 0.17. c) Main effect of reward magnitude manipulation F(1,1160) = 0.38, p = 0.54. d) main effect of reward magnitude manipulation F(1,1172) = 0.31, p = 0.58. e) Main effect of reward magnitude manipulation F(1,1145) = 1.85, p = 0.17. Main effect of reward magnitude manipulation F(1,1160) = 0.38, p = 0.54.

**Incorporating choice history:**

Previous work^3^ has found that when choice history is not modelled, asymmetrical learning effects may be a statistical bias due to higher preservation tendencies (i.e., rats repeating the same choice, irrespective of the outcome). We therefore ran a separate model with a choice history component added. Including this choice history component improved the overall model fit (Figure S9m), suggesting that choice history does capture more variance in the data. However, the asymmetrical learning effects reported in Figure 1f-i (main text) between sex groups remained, suggesting that these results these results may not entirely be due to differences in perseveration tendencies.

In this choice history model, in addition to the four parameters capturing asymmetrical learning and forgetting rates, we added another two parameters to capture 1) the preservation tendencies of each rat (φ) where a higher positive value indicated greater likelihood to repeat previous choice irrespective of the outcome, and 2) choice history decay (τ), where a higher value indicated faster choice history decay and therefore less influence of the choice histories. These were added to the equations below as per^3^:

$Pc(t)= \frac{1}{1+exp(-\beta\left( Qc\left( t \right)-Qu\left( t \right) \right)-\varphi\left( Cc\left( t \right)-Cu\left( t \right) \right))}$ (Equation S1)

Where Pc(t) is the probability of the choice at time t, Qc(t) is the value of the chosen action at time t, Qu(t) is the value of the unchosen action at time t, Cc(t) is the choice at time t and Cu(t) is the unchosen choice at time t. The choice trace was calculated as the following:

$C_{c}\left( t+1 \right)=(C_{c}\left( t \right)+ \tau*(1-C_{c}\left( t \right))$ (Equation S2)

$C_{u}\left( t+1 \right)=(C_{u}\left( t \right)+ \tau*(1-C_{u}\left( t \right))$ (Equation S3)

We also computed the model fit score using leave-one-out cross-validation with pareto-smoothed importance sampling (psis-loo; Figure S9m). The lower the psis-loo score, the better the model fit.


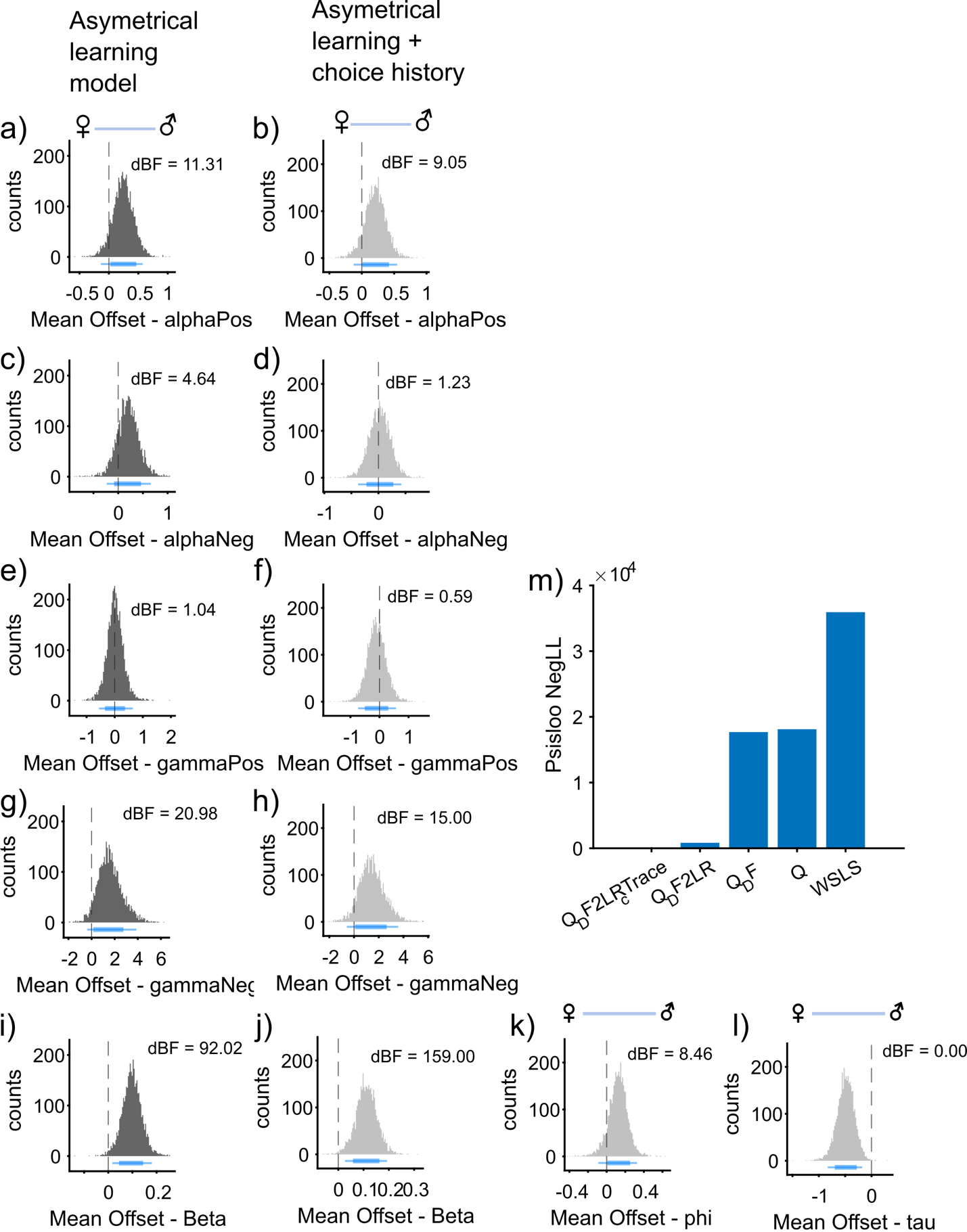


**Figure S9.** *Influence of adding choice history to asymmetrical learning effects.* **a-j)** Posterior densities from the hyperparameter of sex group differences from the asymmetrical value updating model (darker, left) and asymmetrical learning + choice history model (lighter, right). Rightward shifts (above 0) indicate higher parameter values for males compared to females, and the opposite for leftward (below 0) values. Horizontal lines below (blue) represent 80-95% highest density interval. Strength of the rightward skew was quantified by the directed Bayes Factor (dBF) where dBF of 11.31 as in **a** means that males are 11.31 times more likely to have a higher parameter than a lower value compared to females. Overall, we find that the dBF does reduce when using the choice model, however, the overall differences in asymmetrical learning between sex groups still remain. **m)** The model fit scores using pareto-smoothed importance sampling (psis-looNegLL). We found that the model with the choice history component (QDF2LRcTrace) fit the data better than the model without this (QDF2LR). For comparison we also fit a model with just one learning rate and one forgetting rate (irrespective of the outcome; QDF), a standard Q-value with just one learning rate (Q) and a win-stay-lose-shift (WSLS) model. **k)** Males have a higher φ value, indicating they are more likely to repeat the previous choice irrespective of the outcome, compared to females. This result is consistent with males having a higher WS and LS probability than females (Figure 1; main text). **l)** Females have a higher τ value, indicating that females decay the influence of previous choices more so than males.


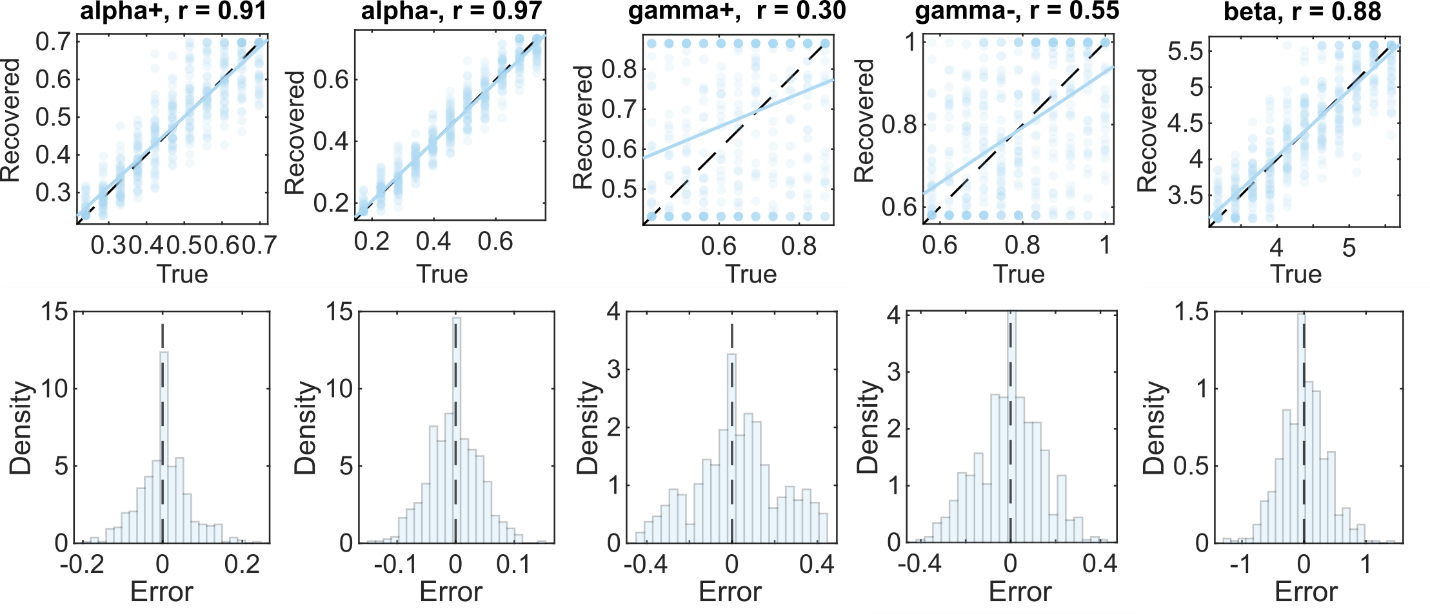


***Figure S10*.** *Parameter recovery from the five-parameter asymmetrical learning model.* Top panel shows the true and recovered parameter estimates, and their correlations as r-value. The bottom panel shows the density function of the recover, where the peak at error of 0 indicates good recovery performance. Given the large number of free parameters in this model, we used a grid-based, controlled refit. For each parameter, the empirical minimum and maximum across rats defined its range. Within that range, evenly spaced grid points were sampled (≈0.1 steps for rates on [0,1]). For each grid point, 50 independent datasets of 500 trials were simulated from the same probabilistic structure of the dynaPRL task. During the simulations, the target parameter was set to the grid value, while the other four parameters were fixed at the midpoint of their empirical ranges. Refit then estimated one parameter at a time, keeping the other four fixed to their generating values; the free parameter’s lower/upper bounds are the empirical min/max. We used the same recovery procedure as for the empirical data.


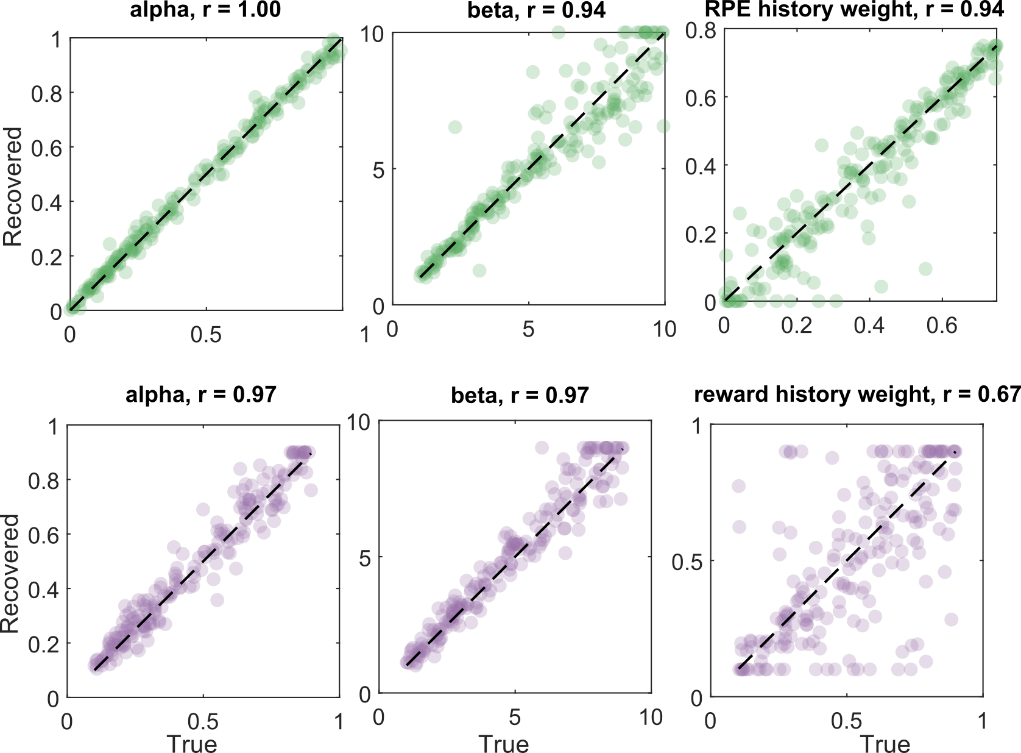


***Figure S11.*** *Parameter recovery of the average reward prediction error (avgRPE-RL) model (top panel, green) and the global reward state (GRS) model (bottom panel, purple).* We ran the same probabilistic conditions as the dynaPRL task for the two models separately. There was a total of 200 simulations over 1000 trials per simulation. In each simulation, the three parameter values were chosen at random, with the same bounds and model fitting procedure as in the recovery of the empirical data. We overall find a positive correlation between the recovered and true parameters.

**References**

1. Trepka, E. *et al.* Entropy-based metrics for predicting choice behavior based on local response to reward. *Nat. Commun.* **12**, 6567 (2021).

2. Woo, J. H. *et al.* Mechanisms of adjustments to different types of uncertainty in the reward environment across mice and monkeys. *Cogn. Affect. Behav. Neurosci.* **23**, 600–619 (2023).

3. Sugawara, M. & Katahira, K. Dissociation between asymmetric value updating and perseverance in human reinforcement learning. *Sci. Rep.* **11**, 3574 (2021).
